# Reduced neutralising antibody responses against emerging 2025/26 influenza A(H1N1)pdm09 subclade D.3.1 and A(H3N2) subclade K viruses among healthcare workers, Finland, August to October 2025

**DOI:** 10.2807/1560-7917.ES.2026.31.6.2600094

**Published:** 2026-02-12

**Authors:** Niina Ikonen, Anu Haveri, Erika Lindh, Oona Liedes, Saimi Vara, Sari H Pakkanen, Anu Kantele, Tea Nieminen, Veli-Jukka Anttila, Hanna Välimaa, Merit Melin, Carita Savolainen-Kopra, Hanna Nohynek

**Affiliations:** 1 Finnish Institute for Health and Welfare, Department of Public Health, Helsinki, Finland; 2 Meilahti Vaccine Research Center MeVac, Department of Infectious Diseases, Helsinki University Hospital and University of Helsinki, Helsinki, Finland; 3 University of Helsinki, Department of Internal Medicine, Helsinki, Finland; 4 HUS New Children’s Hospital, Helsinki University Hospital, Helsinki, Finland; * These authors contributed equally to this work and share first authorship.

**Keywords:** A(H1N1)pdm09, A(H3N2), subclade K, neutralising antibodies, seasonal influenza vaccine, healthcare workers

## Abstract

During autumn 2025, drifted influenza A(H3N2) subclade K and A(H1N1)pdm09 subclade D.3.1. and D.3.1.1 viruses were detected in Finland. We assessed antibody responses against 2024/25 vaccine and 2025/26 epidemic influenza A strains among 46 Finnish healthcare workers before and after influenza vaccination with the 2024/25 vaccine; this vaccine included identical A(H1N1)pdm09 but different A(H3N2) strains compared with the 2025/26 vaccine. Neutralising antibody responses were markedly reduced against the A(H3N2) subclade K virus and titres for certain A(H1N1)pdm09 strains were reduced.

In Finland, as in other European Union/European Economic Area (EU/EEA) countries [1], the 2025/26 influenza season started earlier than in the previous season. To assess immunity against circulating strains, we explored antibody responses to the 2024/25 northern hemisphere seasonal influenza vaccine (SIV) [2] in a cohort of Finnish healthcare workers (HCWs) and compared neutralising antibody levels against influenza A viruses included in the vaccine with responses to selected epidemic virus strains from the 2025/26 season. The influenza A(H1N1)pdm09 strain for the 2025/26 northern hemisphere SIV remained unchanged from the 2024/25 season (A/Victoria/4897/2022), whereas the influenza A(H3N2) vaccine virus was updated for the 2025/26 season [3].

## Genetic characterisation of influenza A(H1N1)pdm09 and A(H3N2) viruses in Finland in 2025/26

As a part of virological surveillance of influenza and to monitor changes of epidemic viruses, we selected a subset of influenza A-positive samples from clinical microbiology laboratories for genetic characterisation by whole genome sequencing; the selection was based on geographical origin and temporal distribution.

We used the Chemagic Viral300 DNA/RNA Kit for nucleic acid extraction and generated amplicons of the viral segments using a one-step reverse transcription PCR with influenza-specific primers [4]. Sequencing libraries were prepared using the Nextera XT DNA Library preparation kit and sequenced on the NextSeq 2000 system and the P1 300-cycle reagent kit (all by Illumina, United States). Assembly of influenza A genomes was performed using the CDC IRMA (v1.3.1) pipeline [5]. We constructed phylogenetic trees using MEGA v12 with the maximum-likelihood method and 1,000 bootstrap replicates. Reference influenza virus sequences were obtained from the Global Initiative on Sharing All Influenza Data (GISAID) and are listed in Supplementary Tables S1 and S2.

**Figure 1:**
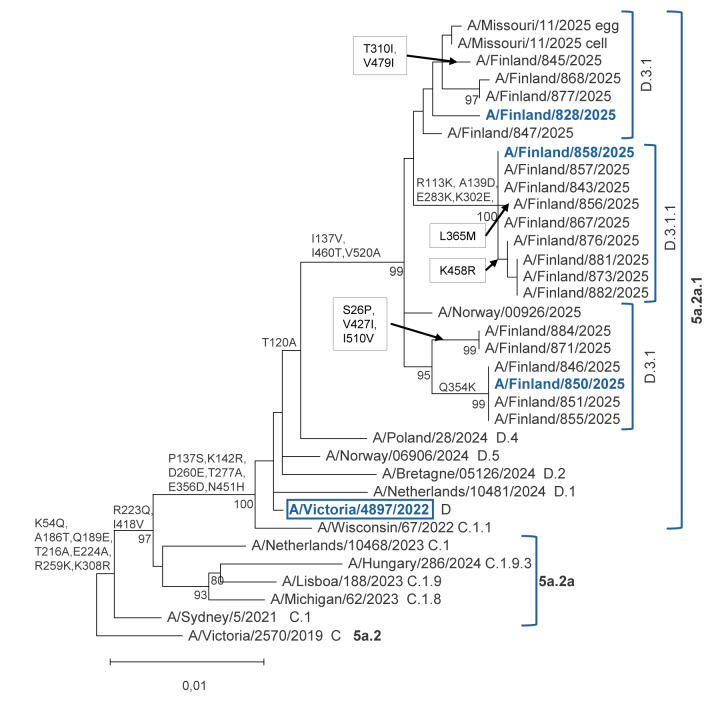
Phylogenetic analysis of haemagglutinin sequences of influenza A(H1N1)pdm09 viruses from surveillance data, Finland, 2025/26 (n = 20)

Between August and November 2025, we sequenced a total of 20 influenza A(H1N1)pdm09 and 31 A(H3N2) viruses. Of the A(H1N1)pdm09) viruses, 11 belonged to subclade D.3.1 and nine to D.3.1.1., four of which carried neuraminidase mutations I223V and S247N associated with oseltamivir resistance (Figure 1). In Supplementary Figure S1, we additionally append a phylogenetic analysis of the neuraminidase gene of influenza A(H1N1)pdm09 viruses from Finnish surveillance data, including the identified mutations.

All sequences included in the phylogenetic tree were 1,650 nt long. The tree was constructed using the maximum likelihood method with Mega software version 12 and with 1,000 bootstrap replicates. Bootstrapping values ≥ 80 are shown. Blue font: the viruses used in microneutralisation tests; blue box: vaccine strain for the 2024/25 and 2025/26 seasons; boxes with arrows represent the location of amino acid substitutions.

**Figure 2:**
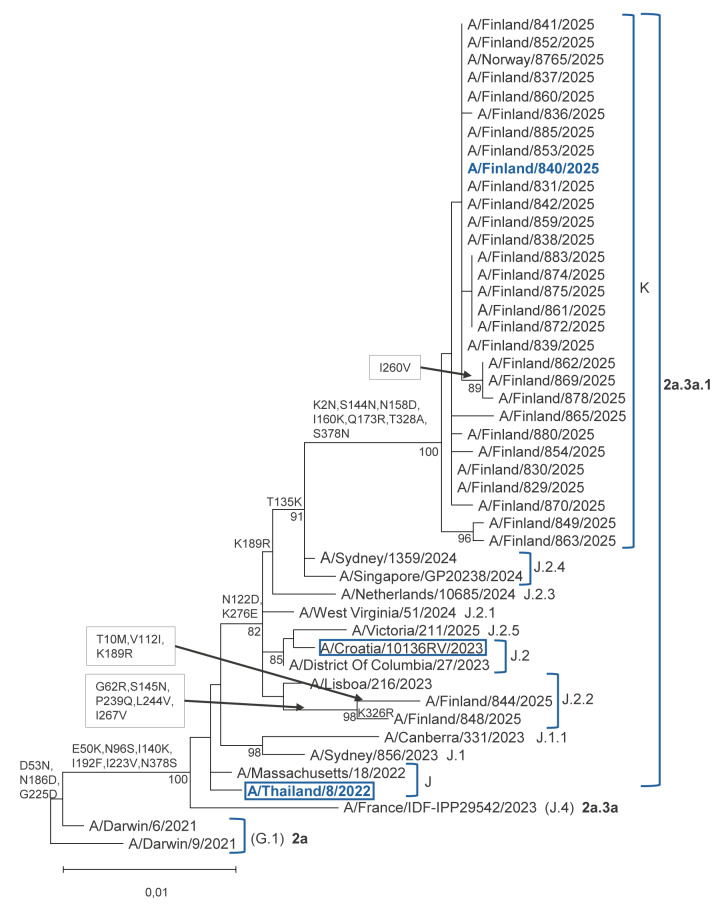
Phylogenetic analysis of haemagglutinin sequences of influenza A(H3N2) viruses from surveillance data, Finland, 2025/26 (n = 31)

Of the 31 influenza A(H3N2) viruses, 29 belonged to subclade K and two to subclade J.2.2 (Figure 2). In Finland, infections caused by subclade K viruses have been detected since August 2025.

All sequences included in the phylogenetic tree were 1,653 nt long. The tree was constructed using the maximum likelihood method with Mega software version 12 and with 1,000 bootstrap replicates. Bootstrapping values ≥ 80 are shown. Blue font: the viruses used in microneutralisation tests; blue boxes: the vaccine strains for season 2024/25 (A/Thailand/8/2022) and 2025/26 (A/Croatia/10136RV/2023); boxes with arrows represent the location of amino acid substitutions.

## Influenza A(H1N1)pdm09 and A(H3N2) antigenic characterisation

We used microneutralisation assay to identify antigenic differences between vaccine viruses and season 2025/26 circulating influenza A viruses. The assay was performed as previously described [6,7] with the labelled antibody diluted 1:6,000 and MDCK-SIAT1 cells used for A(H3N2) viruses. Viruses selected for antigenic characterisation represented genetically different epidemic strains circulating in Finland early in the season 2025/26. Influenza A(H1N1)pdm09 subclade D.3.1 and D.3.1.1 viruses were neutralised very well with egg-propagated A/Victoria/4897/2022 ferret antisera (Table 1).

**Table 1:** Microneutralisation titres of ferret post-infection antisera raised against influenza A(H1N1)pdm09 isolates generated from samples with collection dates in 2025, Finland, August–October 2025 (n = 5)

	A/Michigan/45/2015, egg	A/Victoria/2570/2019, egg	IVR-238 (A/Victoria/4897/2022), egg	Clade or subclade	Passage details^a^	Sample date
Reference antigens						
A/Michigan/45/2015^b^	10,240	40	1,280	6B.1	E6M2	NA
A/Victoria/2570/2019^c^	640	10,240	40,960	5a.2	E5M1	NA
IVR-238 (A/Victoria/4897/2022)^d^	320	5,120	81,920	D	E3D6E2M1	NA
Test antigen						
A/Finland/828/2025	1,280	20,480	163,840	D.3.1	M2	29 Aug 2025
A/Finland/847/2025	640	40,960	≥ 327,680	D.3.1	M2	1 Oct 2025
A/Finland/850/2025	640	10,240	81,920	D.3.1	M2	3 Oct 2025
A/Finland/857/2025	1,280	20,480	163,840	D.3.1.1	M3	9 Oct 2025
A/Finland/858/2025	1,280	20,480	163,840	D.3.1.1	M3	12 Oct 2025

D: influenza vaccine reagent-designated cell-grown virus; E: egg-grown virus; M: Madin−Darby canine kidney cell-grown virus; NA: not applicable.

^a^ Passage details include whether the vaccine strains were egg- or cell-grown, and a number indicating the number of passages.

^b^ Vaccine strain, northern-hemisphere season 2017/18 and 2018/19.

^c^ Vaccine strain, northern-hemisphere season 2021/22 and 2022/23.

^d^ Vaccine strain, northern-hemisphere season 2023/24, 2024/25 and 2025/26.

^d^ Vaccine strain, northern-hemisphere season 2022/23 and 2023/24.

Each column represents a ferret antiserum raised to a particular virus and each row represents either a reference antigen virus (to which an antiserum was raised) or a test antigen virus representing influenza virus isolates from Finland. D, D.3.1 and D.3.1.1 represent the A(H1N1)pdm09 subclade nomenclature.

Influenza A/Finland/840/2025 virus, which belongs to A(H3N2) subclade K, showed a 256-fold reduction for neutralisation with ferret antisera raised against egg-propagated A/Thailand/8/2022. Reduction detected against egg-propagated A/Croatia/10136RV/2023 was in line with recently reported antigenic characterisations [8-10] (Table 2).

**Table 2:** Microneutralisation titres of ferret post-infection antisera raised against influenza A(H3N2) A/Finland/840/2025 isolate generated from sample with collection date in 2025, Finland, September 2025 (n = 1)

	A/Darwin/9/2021, egg	A/Thailand/8/2022, egg	A/Croatia/10136RV/2023, egg	Clade or subclade	Passage details^a^	Sample date
Reference antigens						
A/Darwin/9/2021^b^	2,560	640	640	2a	E7S2	NA
A/Thailand/8/2022^c^	2,560	2,560	2,560	J	E3E1S1	NA
A/Croatia/10136RV/2023^d^	ND	ND	ND	J.2	ND	NA
Test antigen						
A/Finland/840/2025	40	10	40	K	S2	16 Sep 2025

E: egg-grown virus; NA: not applicable; ND: not done; S: Madin−Darby canine kidney cell-SIAT1 cell-grown virus.

J, J.2 and K represent the A(H3N2) subclade nomenclature.

^a^ Passage details include whether the vaccine strains were egg- or cell-grown, and a number indicating the number of passages.

^b^ Vaccine strain, northern-hemisphere season 2022/23 and 2023/24.

^c^ Vaccine strain, northern-hemisphere season 2024/25.

^d^ Vaccine strain, northern-hemisphere season 2025/26.

Each column represents a ferret antiserum raised to a particular virus and each row represents either a reference antigen virus (to which an antiserum was raised) or a test antigen virus representing influenza virus isolates from Finland.

## Monitoring neutralising antibody response after influenza vaccination in a cohort of healthcare workers

To analyse immunity to seasonal influenza, we collected serum samples from HCWs who received the SIV from October to December 2024 as part of their routine annual vaccination. We included 46 HCWs (10 male, 36 female) from Helsinki University Hospital, median age 45 years (range: 23–63), in the analysis of the 2024/25 cohort. Written informed consent was provided by all participants. All participants were vaccinated through occupational health services with the tetravalent, non-adjuvanted 2024/25 SIV in autumn 2024. Serum samples collected before vaccination (day 0) and at a median of 36 days (range: 28–78) after vaccination were analysed in this study.

We tested these serum samples by microneutralisation assays for the presence of neutralising antibodies against the influenza A 2024/25 vaccine viruses and selected influenza virus isolates from the 2025/26 season. The SIV strains for the northern hemisphere 2024/25 were A/Victoria/4897/2022 (A(H1N1)pdm09, subclade D) and A/Thailand/8/2022 (A(H3N2), subclade J). The following Finnish strains from the 2025/26 season were included in the analyses: A/Finland/840/2025 (A(H3N2), subclade K), A/Finland/828/2025 (A(H1N1)pdm09, subclade D.3.1), A/Finland/850/2025 (A(H1N1)pdm09, subclade D.3.1), and A/Finland/858/2025 (A(H1N1)pdm09, subclade D.3.1.1).

For statistical analyses, serum specimens with titres < 10 were assigned a titre value of 5. We calculated the geometric mean titres (GMT) with 95% confidence intervals (CIs) for each virus. Statistical significance of differences was estimated using Friedman test followed by Dunn’s multiple comparisons test or Wilcoxon matched-pairs signed-rank test, with a significance level of p < 0.05.

For all virus strains tested, there was a significant (p < 0.0001) increase in the GMTs of the neutralising antibody response 5 weeks after SIV vaccination (Table 3). The GMTs’ fold change was 1.6–1.9 and 1.8–2.3, respectively, for influenza A(H1N1)pdm09 and A(H3N2) viruses.

**Table 3:** Geometric mean titres against influenza A(H1N1)pdm09 and A(H3N2) viral strains measured by microneutralisation test before and after vaccination of healthcare workers with 2024/25 quadrivalent influenza vaccine, Finland, October 2024–January 2025 (n = 46)

Influenza virus strain	Clade	Subclade	Geometric mean titres (95% CI)		Fold change
			Day 0	Day 36	
A(H1N1)pdm09					
IVR-238 (A/Victoria/4897/2023)^a^	5a.2a.1	D	274 (204–367)	438 (333–577)	1.6
A/Finland/828/2025	5a.2a.1	D.3.1	185 (135–253)	333 (235–471)	1.8
A/Finland/850/2025	5a.2a.1	D.3.1	73.3 (52.7–102)	133 (91.7–194)	1.8
A/Finland/858/2025	5a.2a.1	D.3.1.1	189 (147–242)	364 (257–515)	1.9
A(H3N2)					
A/Thailand/8/2022^b^	2a.3a.1	J	148 (112–197)	336 (252–448)	2.3
A/Finland/840/2025	2a.3a.1	K	16.7 (13.0–21.5)	30.0 (23.4–38.5)	1.8

CI: confidence interval.

^a^ Vaccine strain, northern-hemisphere seasons 2023/24, 2024/25 and 2025/26.

^b^ Vaccine strain, northern-hemisphere season 2024/25.

One dose of non-adjuvanted quadrivalent seasonal influenza vaccine 2024/25 was administered intramuscularly to Finnish healthcare workers. Day 0 refers to serum samples collected before vaccination (median: 1 day, range: 0–9). J and K represent the influenza A(H3N2) subclade nomenclature, while D, D.3.1 and D.3.1.1 represent the A(H1N1)pdm09 subclade nomenclature.

Neutralising antibody titres against influenza A(H1N1)pdm09 subclade D.3.1. strain A/Finland/850/2025 possessing haemagglutinin mutation Q354K, were markedly lower (p < 0.0001) both pre- (3.7-fold reduction) and post-vaccination (3.3-fold reduction) than against the vaccine strain A/Victoria/4897/2022 (Table 3). The A(H1N1)pdm09 titres presented in Table 3 are visualised in Supplementary Figure S2. GMTs for A/Finland/828/2025 (subclade D.3.1) and A/Finland/858/2025 (subclade D.3.1.1) were less than twofold lower than the vaccine strain. Pre- and post-vaccination GMTs for A/Finland/850/2025 were lower than those for A/Finland/828/2025 and A/Finland/858/2025 (p < 0.0001). This finding was consistent with lower seroprotection rates, considering a microneutralisation titre of 160 equivalent to a haemagglutination inhibition titre of 40 [11], appended in Supplementary Table S3.

The GMTs against influenza A/Finland/840/2025 (subclade K) were significantly lower (p < 0.0001) than against the homologous A(H3N2) 2024/25 vaccine strain A/Thailand/8/2022, with fold reductions of 8.9 before and 11.2 after SIV vaccination (p < 0.0001) (Table 3). In Supplementary Figure S3, the A(H3N2) titres shown in Table 3 are visualised.

## Discussion

While the increased prevalence of influenza A(H3N2) subclade K viruses extended the season in Australia and New Zealand [8], season 2025/26 influenza activity in the EU/EEA increased 3–4 weeks earlier than in the previous two seasons [1]. Drifted influenza A(H3N2) subclade K viruses have predominated in Europe [1], and drifted A(H1N1)pdm09 D.3.1 and D.3.1.1 viruses have also been reported [12].

In the 2025/26 season, influenza A(H3N2) viruses co-circulate with A(H1N1)pdm09 subclades D.3.1 and D.3.1.1 in Finland. We have detected a cluster of subclade D.3.1.1 strains carrying neuraminidase mutations linked to oseltamivir resistance. Neuraminidase mutation S247N has previously been reported elsewhere in Europe [13]. These findings highlight the need to monitor whether such viruses become more prevalent.

New antigenic influenza A(H3N2) clusters appear on average every 3.3 years [14]. Seven amino acid locations have been shown to be responsible for the major antigenic changes in A(H3N2) viruses [15]. Subclade K has a substantially higher number of mutations compared with the evolution of A(H3N2) viruses observed in previous seasons. These mutations include K2N, T135K, S144N(+CHO), N158D, I160K, Q173R, K189R, T328A and S378N substitutions in the haemagglutinin gene compared with the A/Croatia/10136RV/2023 vaccine strain for the 2025/26 season [16,17].

The antigenic characterisation using microneutralisation test demonstrated poor reactivity for K virus with post-infection ferret antisera against both 2024/25 and 2025/26 A(H3N2) vaccine viruses. The reduction was in line with recently reported antigenic characterisations by haemagglutination inhibition tests [8-10]. Following the update of the A(H3N2) vaccine component for 2025/26 to A/Croatia/10136RV/2023, it is not unexpected that neutralising antibody titres against the K virus were substantially lower than against the 2024/25 vaccine virus, A/Thailand/8/2022.

In contrast with antigenic characterisations, early estimates of the current seasonal influenza vaccine effectiveness have indicated that the SIV provide moderate protection against influenza A(H3N2) infection at least among children and working age adults [1,9,10,12].

Influenza A(H1N1)pdm09 viruses have undergone genetic changes from the A/Victoria/4897/2023 strain present in the vaccine. Our results indicate lower seroprotection and significantly lower titres for the D.3.1. strain A/Finland/850/2025 possessing the haemagglutinin mutation Q354K compared with the vaccine virus. However, a strong pre-existing immunity against influenza A(H1N1)pdm09 viruses among HCWs as a result of sequential SIV vaccinations or natural infections with influenza A(H1N1)pdm09 viruses are likely to confer high levels of cross-reactive antibodies.

A key limitation of this study is that the analysed sera were collected during the previous influenza season. Therefore, direct comparisons for the 2025/26 season are limited to influenza A(H1N1)pdm09 virus, which was unchanged from the 2024/25 vaccine. In addition, the small number of HCWs may introduce random selection bias. However, the cohort represents a defined population routinely recommended for annual influenza vaccination, namely healthcare personnel, and is therefore a relevant group. It is also noted that immune responses to vaccination and infection may differ in children and older adults. 

Our findings contribute to the assessment of neutralising antibody responses induced by prior SIV against currently circulating viruses. Such data support interpretation of population-level immunity and may inform early assessments of the potential disease burden during the ongoing season. Furthermore, relating observed amino acid substitutions to changes in antibody neutralisation may assist in the characterisation of emerging strains relevant for vaccine strain selection. Antigenic predictions based on genetic data alone do not always correlate with serological outcomes, underscoring the continued need for human serological data within routine influenza surveillance.

## Conclusion

Our serological data suggest a possible suboptimal neutralising antibody response against influenza A(H3N2) K virus variants and indicate that the A(H3N2) component of SIV needs to be updated frequently to better match the drifted subclade. In addition, drifted influenza A(H1N1)pdm09 strains require further attention. Despite imperfect vaccine performance and occasional suboptimal antigenic matching, influenza vaccination remains beneficial both at the individual and population level. Influenza vaccination is strongly encouraged for HCWs and individuals in risk groups to strengthen immunity, reduce disease burden and limit epidemic spread.

## Data Availability

All sequence data are publicly available on GISAID website www.gisaid.org. At the outset of the trial, data-sharing provisions were not included in the informed consent documents signed by participants. In accordance with ethics and institutional policies, we are not authorised to release individual-level or pseudo-anonymised datasets to the public. To protect participant privacy, only de-identified, aggregated group-level values (without background or individual-level information) are available. These data can be requested from the corresponding author.

## Supplementary Data

422000 Supplement
