# Supplementary material for: Reduced neutralising antibody responses against emerging 2025/26 influenza A(H1N1)pdm09 subclade D.3.1 and A(H3N2) subclade K viruses among healthcare workers, Finland, August to October 2025
Source: Euro Surveill. 2026 Feb 12;31(6):2600094. doi: 10.2807/1560-7917.ES.2026.31.6.2600094 (PMC12905531; doi:10.2807/1560-7917.ES.2026.31.6.2600094)
Supplement: Supplement [file 26-00094_HAVERI_Supplement.pdf]

Supplementary material

Reduced neutralising antibody responses against emerging 2025/26 influenza A(H1N1)pdm09 subclade D.3.1 and A(H3N2) subclade K viruses among healthcare workers, Finland, August–October 2025

This supplementary material is hosted by *Eurosurveillance* as supporting information alongside the article 'Reduced neutralising antibody responses against emerging 2025/26 influenza A(H1N1)pdm09 subclade D.3.1 and A(H3N2) subclade K viruses among healthcare workers, Finland, August–October 2025', on behalf of the authors, who remain responsible for the accuracy and appropriateness of the content. The same standards for ethics, copyright, attributions and permissions as for the article apply. Supplements are not edited by *Eurosurveillance* and the journal is not responsible for the maintenance of any links or email addresses provided therein.

Supplementary Table S1. Origin of the sequence information of influenza A(H1N1)pdm09 viruses included in the phylogenetic analysis.

Isolate name	GISAID accession number	Country	Collection date	Originating laboratory	Submitting laboratory	Authors
A/Victoria/2570/2019 egg	EPI_ISL_417210	Australia	2019-11-22	Alfred Hospital	WHO Collaborating Centre for Reference and Research on Influenza	Deng,Y-M; Iannello,P; Lau,H; Todd,A; Spirason,N; Moselen,J; Komadina, N.
A/Sydney/5/2021 egg	EPI_ISL_12109635	Australia	2021-10-16	WHO Collaborating Centre for Reference and Research on Influenza	Centers for Disease Control and Prevention Influenza Division	.
A/Lisboa/188/2023	EPI_ISL_19334296	Portugal	2023-11-22	Instituto Nacional de Saude (INSA) National Influenza Reference Laboratory	Crick Worlwide Influenza Centre	
A/Hungary/286/2024	EPI_ISL_19685707	Hungary	2024-11-05	Central Veterinary Institute Budapest	Crick Worlwide Influenza Centre	
A/Michigan/62/2023	EPI_ISL_19175842	United States	2023-10-16	Michigan Department of Community Health	Centers for Disease Control and Prevention	
A/Netherlands/10468/2023	EPI_ISL_18044584	Netherlands	2023-03-13	National Institute for Public Health and the Environment (RIVM)	Crick Worldwide Influenza Centre	

A/Wisconsin/67/2022 cell	EPI_ISL_15928563	United States	2022-10-25	Wisconsin State Laboratory of Hygiene	Centers for Disease Control and Prevention	
A/Norway/06906/2024	EPI_ISL_19497280	Norway	2024-09-23	Norwegian Institute of Public Health	Norwegian Institute of Public Health	Bragstad, K; Hungnes, O; Madsen, MP; Rohringer, A; Riis, R; Knutsen, MF
A/Netherlands/10481/2024	EPI_ISL_19252612	Netherlands	2024-02-12	National Institute for Public Health and the Environment (RIVM)	Crick Worldwide Influenza Centre	
A/Victoria/4897/2022 egg	EPI_ISL_17072386	Australia	2022-10-02	WHO Collaborating Centre for Reference and Research on Influenza	Nationla Institute for Biological Standards and Control (NIBSC)	Nicolson, C
A/Bretagne/05126/2024	EPI_ISL_19406762	France	2024-03-29	Institut Pasteur	Crick Worldwide Influenza Centre	
A/Poland/28/2024	EPI_ISL_19204767	Poland	2024-03-04	National Institute of Public Health - National Institute of Hygiene	Crick Worldwide Influenza Centre	
A/Finland/848/2025	EPI_ISL_20247823	Finland	2025-10-02	Helsingin-Uusimaa Labotariokeskus	Finnish Institute for Health and Welfare, THL	Ikonen, N.; Lindh, E.
A/Finland/844/2025	EPI_ISL_20247819	Finland	2025-09-20	Finnish Institute for Health and Welfare, THL	Finnish Institute for Health and Welfare, THL	Ikonen, N.; Lindh, E.
A/Finland/858/2025	EPI_ISL_20247833	Finland	2025-10-12	Helsingin-Uusimaa Labotariokeskus	Finnish Institute for Health and Welfare, THL	Ikonen, N.; Lindh, E.
A/Finland/856/2025	EPI_ISL_20247831	Finland	2025-10-12	Helsingin-Uusimaa Labotariokeskus	Finnish Institute for Health and Welfare, THL	Ikonen, N.; Lindh, E.
A/Finland/843/2025	EPI_ISL_20247818	Finland	2025-09-23	Helsingin-Uusimaa Labotariokeskus	Finnish Institute for Health and Welfare, THL	Ikonen, N.; Lindh, E.
A/Finland/857/2025	EPI_ISL_20247832	Finland	2025-10-09	Helsingin-Uusimaa Labotariokeskus	Finnish Institute for Health and Welfare, THL	Ikonen, N.; Lindh, E.
A/Finland/867/2025	EPI_ISL_20287950	Finland	2025-10-23	Nordlab Oulu	Finnish Institute for Health and Welfare, THL	Ikonen, N.; Lindh, E.
A/Finland/876/2025	EPI_ISL_20287959	Finland	2025-11-22	SataDiag	Finnish Institute for Health and Welfare, THL	Ikonen, N.; Lindh, E.
A/Finland/873/2025	EPI_ISL_20287956	Finland	2025-11-18	Helsingin-Uusimaa Labotariokeskus	Finnish Institute for Health and Welfare, THL	Ikonen, N.; Lindh, E.

A/Finland/881/2025	EPI_ISL_20287963	Finland	2025-11-24	Helsingin-Uusimaa Labotariokeskus	Finnish Institute for Health and Welfare, THL	Ikonen, N.; Lindh, E.
A/Finland/882/2025	EPI_ISL_20287964	Finland	2025-11-28	Helsingin-Uusimaa Labotariokeskus	Finnish Institute for Health and Welfare, THL	Ikonen, N.; Lindh, E.
A/Finland/855/2025	EPI_ISL_20247830	Finland	2025-09-25	Nordlab Oulu	Finnish Institute for Health and Welfare, THL	Ikonen, N.; Lindh, E.
A/Finland/851/2025	EPI_ISL_20247826	Finland	2025-09-24	Ita-Suomen Labotariokeskus	Finnish Institute for Health and Welfare, THL	Ikonen, N.; Lindh, E.
A/Finland/850/2025	EPI_ISL_20247825	Finland	2025-10-03	Nordlab Kainuu	Finnish Institute for Health and Welfare, THL	Ikonen, N.; Lindh, E.
A/Finland/846/2025	EPI_ISL_20247821	Finland	2025-09-28	SataDiag	Finnish Institute for Health and Welfare, THL	Ikonen, N.; Lindh, E.
A/Finland/871/2025	EPI_ISL_20287954	Finland	2025-11-10	Ita-Suomen Labotariokeskus	Finnish Institute for Health and Welfare, THL	Ikonen, N.; Lindh, E.
A/Finland/884/2025	EPI_ISL_20287966	Finland	2025-11-19	Ita-Suomen Labotariokeskus	Finnish Institute for Health and Welfare, THL	Ikonen, N.; Lindh, E.
A/Norway/00926/2025	EPI_ISL_19731580	Norway	2025-01-20	Norwegian Institute of Public Health	Norwegian Institute of Public Health	Bragstad, K; Hungnes, O; Madsen, MP; Rohringer, A; Riis, R; Knutsen, MF
A/Finland/847/2025	EPI_ISL_20247822	Finland	2025-10-01	Finnish Institute for Health and Welfare, THL	Finnish Institute for Health and Welfare, THL	Ikonen, N.; Lindh, E.
A/Finland/828/2025	EPI_ISL_20247803	Finland	2025-08-29	Helsingin-Uusimaa Labotariokeskus	Finnish Institute for Health and Welfare, THL	Ikonen, N.; Lindh, E.
A/Finland/868/2025	EPI_ISL_20287951	Finland	2025-11-15	Helsingin-Uusimaa Labotariokeskus	Finnish Institute for Health and Welfare, THL	Ikonen, N.; Lindh, E.
A/Finland/877/2025	EPI_ISL_20287960	Finland	2025-11-18	SataDiag	Finnish Institute for Health and Welfare, THL	Ikonen, N.; Lindh, E.
A/Finland/845/2025	EPI_ISL_20247820	Finland	2025-09-25	Helsingin-Uusimaa Labotariokeskus	Finnish Institute for Health and Welfare, THL	Ikonen, N.; Lindh, E.
A/Missouri/11/2025 egg	EPI_ISL_20066835	United States	2025-01-30	Missouri Department. of Health & Senior Services	Centers for Disease Control and Prevention	
A/Missouri/11/2025 cell	EPI_ISL_19759016	United States	2025-01-30	Missouri Department. of Health & Senior Services	Centers for Disease Control and Prevention	

Supplementary Table S2. Origin of the sequence information of influenza A(H3N2) viruses included in the phylogenetic analysis.

Isolate name	GISAID accession number	Country	Collection date	Originating laboratory	Submitting laboratory	Authors
A/Darwin/9/2021 egg	EPI_ISL_3801278	Australia	2021-04-17	Royal Darwin Hospital	WHO Collaborating Centre for Reference and Research on Influenza	Deng,Y-M; Iannello,P; Lau,H; Spirason,N; Moselen,J; Aziz,A; Komadina,N.
A/Darwin/6/2021 cell	EPI_ISL_3534319	Australia	2021-03-16	WHO Collaborating Centre for Reference and Research on Influenza	Centers for Disease Control and Prevention	
A/Massachusetts/18/2022 cell	EPI_ISL_16998756	United States	2022-06-04	Massachusetts Department of Public Health	Centers for Disease Control and Prevention	
A/Thailand/8/2022 egg	EPI_ISL_16014504	Thailand	2022-07-11	WHO National Influenza Centre, National Institute of Medical Research (NIMR)	WHO Collaborating Centre for Reference and Research on Influenza	Okada,P; Yuigun,S; Kala,S; Deng,Y-M; Barr,I; Aziz,A
A/Canberra/331/2023	EPI_ISL_19030763	Australia	2023-10-29	Canberra Hospital	WHO Collaborating Centre for Reference and Research on Influenza	Deng,Y-M; Barr,I; Spirason,N; Iannello,P; Lau,H; Dong,X; Chanthalavanh,P; Lay,O; Edwards,S; Hirankitti,A; Dapat,C
A/Sydney/856/2023	EPI_ISL_19085832	Australia	2023-12-09	WHO Collaborating Centre for Reference and Research on Influenza	Crick Worldwide Influenza Centre	
A/France/IDF-IPP29542/2023	EPI_ISL_18949967	France	2023-11-30	Institut Pasteur	Crick Worldwide Influenza Centre	
A/Victoria/211/2025	EPI_ISL_19940075	Australia	2025-01-12	Monash Medical Centre	WHO Collaborating Centre for Reference and	Soppe,S; Rynehart, C; Peck,H; Dapat,C

					Research on Influenza	
A/Croatia/10136RV/2023	EPI_ISL_19085873	Croatia	2023-12-04	Croatian Institute of Public Health	Crick Worldwide Influenza Centre	
A/District Of Columbia/27/2023	EPI_ISL_19175844	United States	2023-12-09	DC Public Health Lab	Centers for Disease Control and Prevention	
A/West Virginia/51/2024	EPI_ISL_19376866	United States	2024-07-04	West Virginia Office of Laboratory Services	Centers for Disease Control and Prevention	
A/Lisboa/216/2023	EPI_ISL_19313759	Portugal	2023-12-15	Instituto Nacional de Saude (INSA)	Crick Worldwide Influenza Centre	
A/Netherlands/10685/2024	EPI_ISL_19767813	Netherlands	2024-12-12	National Institute for Public Health and the Environment (RIVM)	Crick Worldwide Influenza Centre	
A/Sydney/1359/2024	EPI_ISL_19711425	Australia	2024-11-29	Childrens Hospital Westmead	WHO Collaborating Centre for Reference and Research on Influenza	Deng,Y-M; Barr,I; Spirason,N; Wordsworth,R; Lau,H; Dong,X; Chanthalavanh,P; Lay,O; Edwards,S; Hirankitti,A; Dapat,C
A/Singapore/GP20238/2024	EPI_ISL_19871656	Singapore	2024-12-26	Ministry of Health, Singapore	WHO Collaborating Centre for Reference and Research on Influenza	Deng,Y-M; Barr,I; Spirason,N; Wordsworth,R; Lau,H; Dong,X; Chanthalavanh,P; Lay,O; Edwards,S; Hirankitti,A; Dapat,C
A/Norway/8765/2025	EPI_ISL_20205997	Norway	2025-08-14	WHO National Influenza Centre	Crick Worldwide Influenza Centre	
A/Finland/863/2025	EPI_ISL_20287948	Finland	2025-10-22	Helsingin-Uusimaa Labotariokeskus	Finnish Institute for Health and Welfare, THL	Ikonen, N.; Lindh, E.
A/Finland/849/2025	EPI_ISL_20247824	Finland	2025-09-24	Finnish Institute for Health and Welfare, THL	Finnish Institute for Health and Welfare, THL	Ikonen, N.; Lindh, E.
A/Finland/865/2025	EPI_ISL_20287949	Finland	2025-10-25	Helsingin-Uusimaa	Finnish Institute for	Ikonen, N.; Lindh, E.

				Labotariokes kus	Health and Welfare, THL	
A/Finland/880/2025	EPI_ISL_202 87962	Finland	2025-11-24	Helsingin-Uusimaa Labotariokes kus	Finnish Institute for Health and Welfare, THL	Ikonen, N.; Lindh, E.
A/Finland/829/2025	EPI_ISL_202 47804	Finland	2025-09-02	Ita-Suomen Labotariokes kus	Finnish Institute for Health and Welfare, THL	Ikonen, N.; Lindh, E.
A/Finland/854/2025	EPI_ISL_202 47829	Finland	2025-10-06	Helsingin-Uusimaa Labotariokes kus	Finnish Institute for Health and Welfare, THL	Ikonen, N.; Lindh, E.
A/Finland/870/2025	EPI_ISL_202 87953	Finland	2025-11-10	Fimlab Laboratories	Finnish Institute for Health and Welfare, THL	Ikonen, N.; Lindh, E.
A/Finland/830/2025	EPI_ISL_202 47805	Finland	2025-08-31	Nordlab Oulu	Finnish Institute for Health and Welfare, THL	Ikonen, N.; Lindh, E.
A/Finland/878/2025	EPI_ISL_202 87961	Finland	2025-11-19	Helsingin-Uusimaa Labotariokes kus	Finnish Institute for Health and Welfare, THL	Ikonen, N.; Lindh, E.
A/Finland/869/2025	EPI_ISL_202 87952	Finland	2025-11-05	Fimlab Laboratories	Finnish Institute for Health and Welfare, THL	Ikonen, N.; Lindh, E.
A/Finland/862/2025	EPI_ISL_202 87947	Finland	2025-10-21	Nordlab Oulu	Finnish Institute for Health and Welfare, THL	Ikonen, N.; Lindh, E.
A/Finland/885/2025	EPI_ISL_202 87967	Finland	2025-11-20	Ita-Suomen Labotariokes kus	Finnish Institute for Health and Welfare, THL	Ikonen, N.; Lindh, E.
A/Finland/860/2025	EPI_ISL_202 47835	Finland	2025-10-08	SataDiag	Finnish Institute for Health and Welfare, THL	Ikonen, N.; Lindh, E.
A/Finland/853/2025	EPI_ISL_202 47828	Finland	2025-09-22	Fimlab Laboratories	Finnish Institute for Health and Welfare, THL	Ikonen, N.; Lindh, E.
A/Finland/841/2025	EPI_ISL_202 47816	Finland	2025-09-16	Fimlab Laboratories	Finnish Institute for Health and Welfare, THL	Ikonen, N.; Lindh, E.
A/Finland/838/2025	EPI_ISL_202 47813	Finland	2025-09-15	Fimlab Laboratories	Finnish Institute for Health and Welfare, THL	Ikonen, N.; Lindh, E.
A/Finland/859/2025	EPI_ISL_202 47834	Finland	2025-10-06	SataDiag	Finnish Institute for Health and Welfare, THL	Ikonen, N.; Lindh, E.
A/Finland/836/2025	EPI_ISL_202 47811	Finland	2025-09-08	Fimlab Laboratories	Finnish Institute for Health and Welfare, THL	Ikonen, N.; Lindh, E.

A/Finland/842/2025	EPI_ISL_20247817	Finland	2025-09-18	Fimlab Laboratories	Finnish Institute for Health and Welfare, THL	Ikonen, N.; Lindh, E.
A/Finland/839/2025	EPI_ISL_20247814	Finland	2025-09-15	Fimlab Laboratories	Finnish Institute for Health and Welfare, THL	Ikonen, N.; Lindh, E.
A/Finland/831/2025	EPI_ISL_20247806	Finland	2025-09-04	Fimlab Laboratories	Finnish Institute for Health and Welfare, THL	Ikonen, N.; Lindh, E.
A/Finland/837/2025	EPI_ISL_20247812	Finland	2025-09-09	Fimlab Laboratories	Finnish Institute for Health and Welfare, THL	Ikonen, N.; Lindh, E.
A/Finland/840/2025	EPI_ISL_20247815	Finland	2025-09-16	Fimlab Laboratories	Finnish Institute for Health and Welfare, THL	Ikonen, N.; Lindh, E.
A/Finland/852/2025	EPI_ISL_20247827	Finland	2025-10-02	SataDiag	Finnish Institute for Health and Welfare, THL	Ikonen, N.; Lindh, E.
A/Finland/861/2025	EPI_ISL_20247836	Finland	2025-10-13	Helsingin-Uusimaa Labotariokeskus	Finnish Institute for Health and Welfare, THL	Ikonen, N.; Lindh, E.
A/Finland/874/2025	EPI_ISL_20287957	Finland	2025-11-17	Helsingin-Uusimaa Labotariokeskus	Finnish Institute for Health and Welfare, THL	Ikonen, N.; Lindh, E.
A/Finland/875/2025	EPI_ISL_20287958	Finland	2025-11-14	Nordlab Kainuu	Finnish Institute for Health and Welfare, THL	Ikonen, N.; Lindh, E.
A/Finland/883/2025	EPI_ISL_20287965	Finland	2025-11-21	Nordlab Oulu	Finnish Institute for Health and Welfare, THL	Ikonen, N.; Lindh, E.

Supplementary Figure S1. Phylogenetic analysis of the neuraminidase sequences of influenza A(H1N1pdm09) viruses from Finnish surveillance data during 2025/26.

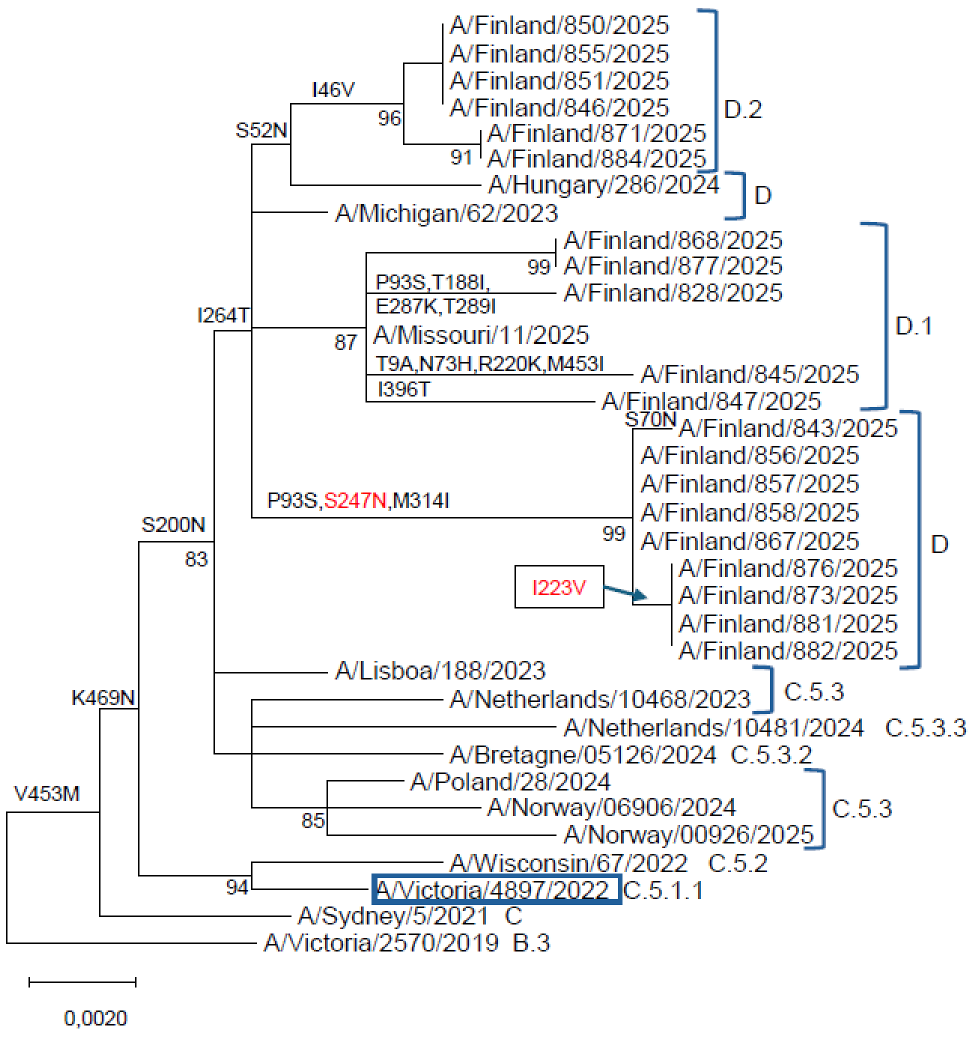

All sequences included in the phylogenetic tree were 1,410 nucleotides long. The tree was constructed using the maximum likelihood method with Mega software version 12 and with 1,000 bootstrap replicates. Bootstrapping values ≥ 80 are shown. Arrowed lines represent the location of amino acid substitutions. Amino acid substitutions associated with oseltamivir resistance are shown in red. The vaccine strain for seasons 2024/25 and 2025/26 is shown in box.

Supplementary Figure S2. Neutralising antibody responses targeting the vaccine antigen 2023/24, 2024/25 and 2025/26 (A/Victoria/4897/2022) and epidemic A(H1N1)pdm09 viruses among Finnish healthcare workers n=46 **A.** pre vaccination (day 0, range 0–9) and **B.** post seasonal influenza vaccination day 36 (range 28–72).

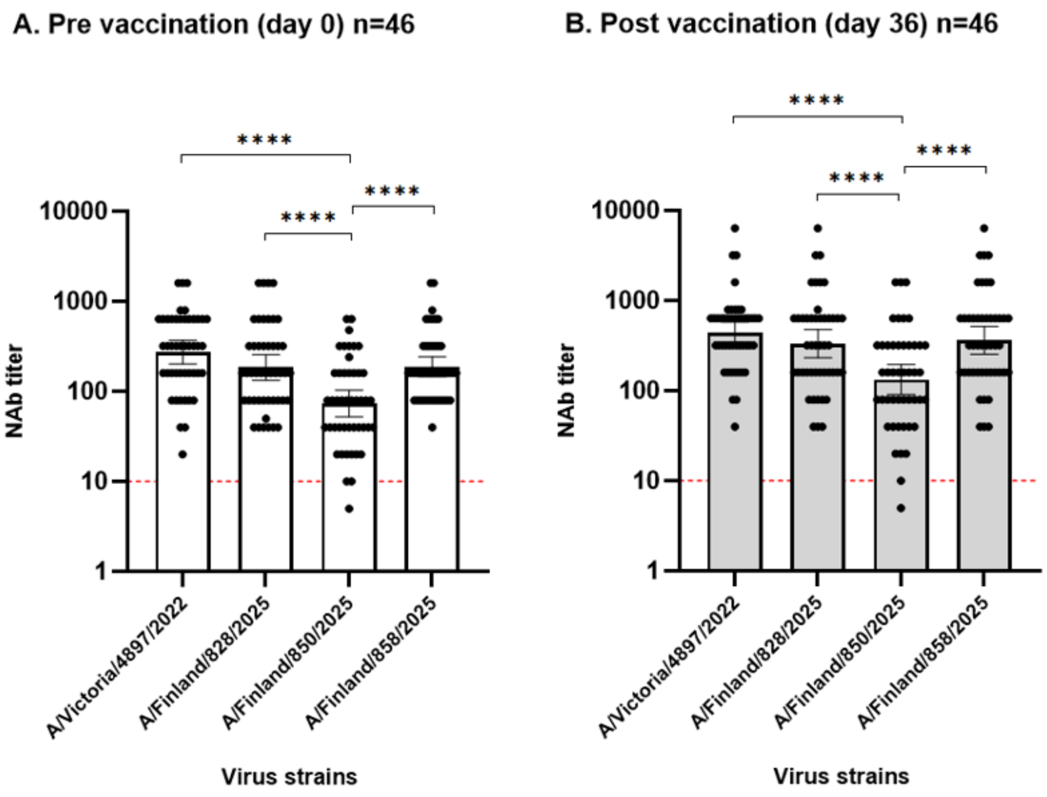

The graphs display geometric mean titres (GMT) and 95% CIs (whiskers). The dashed line indicates the positivity threshold; a titre of 10 or above was considered positive. Only statistically significant differences between virus strains at the same time point are indicated. Comparisons between virus strains were conducted using two-sided Friedman test followed by Dunn's multiple comparisons test.

Supplementary Table S3. Seroprotection rates determined by microneutralisation test before and after vaccination of 46 healthcare workers with seasonal influenza vaccine 2024/25.

Influenza A(H1N1)pdm09 virus strain	Clade	Subclade	Seroprotection rates (n/n)	
			Day 0	Day 36
IVR-238 (A/Victoria/4897/2023)[a]	5a.2a.1	D	37/46	43/46
A/Finland/828/2025	5a.2a.1	D.3.1	29/46	38/46
A/Finland/850/2025	5a.2a.1	D.3.1	16/46	25/46
A/Finland/858/2025	5a.2a.1	D.3.1.1	32/46	40/46

D, D.3.1 and D.3.1.1 represent the A(H1N1)pdm09 subclade nomenclature.

One dose of non-adjuvanted quadrivalent seasonal influenza vaccine 2024/25 was administered intramuscularly to Finnish healthcare workers. Seroprotection rate was defined as the proportion of subjects with a microneutralisation titre ≥ 160. Day 0 refers to serum samples collected before vaccination.

[a] Vaccine strain, northern-hemisphere season 2023/24, 2024/25 and 2025/26.

Supplementary Figure S3. Neutralising antibody responses targeting the vaccine antigen 2024/25 (A/Thailand/8/2022) and epidemic A(H3N2) subclade K virus among Finnish healthcare workers n=46 **A.** pre vaccination (day 0, range 0−9) and **B.** post seasonal influenza vaccination day 36 (range 28−72).

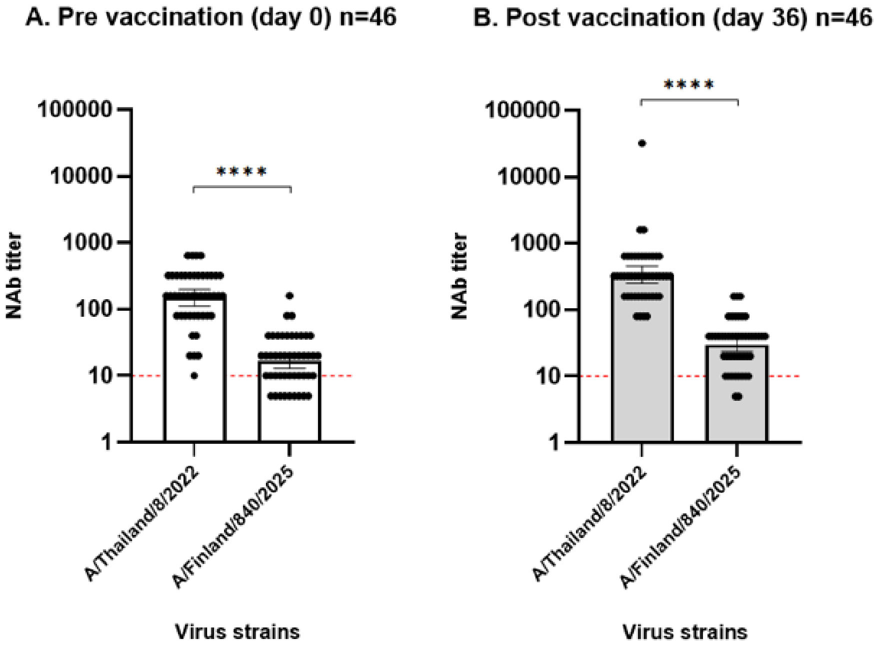

The graphs display geometric mean titres (GMT) and 95% CIs (whiskers). The dashed line indicates the positivity threshold; a titre of 10 or above was considered positive. Only statistically significant differences between virus strains at the same time point are indicated. Comparisons between virus strains were conducted using two-sided Wilcoxon matched-pairs signed-rank test.
